# An Essential Role for Medullary Thymic Epithelial Cells during the Intrathymic Development of Invariant NKT Cells

**DOI:** 10.4049/jimmunol.1303057

**Published:** 2014-02-07

**Authors:** Andrea J. White, William E. Jenkinson, Jennifer E. Cowan, Sonia M. Parnell, Andrea Bacon, Nick D. Jones, Eric J. Jenkinson, Graham Anderson

**Affiliations:** Medical Research Council Centre for Immune Regulation, Institute for Biomedical Research, University of Birmingham, Birmingham B15 2TT, United Kingdom

## Abstract

In the thymus, interactions with both cortical and medullary microenvironments regulate the development of self-tolerant conventional CD4^+^ and CD8^+^ αβT cells expressing a wide range of αβTCR specificities. Additionally, the cortex is also required for the development of invariant NKT (iNKT) cells, a specialized subset of T cells that expresses a restricted αβTCR repertoire and is linked to the regulation of innate and adaptive immune responses. Although the role of the cortex in this process is to enable recognition of CD1d molecules expressed by CD4^+^CD8^+^ thymocyte precursors, the requirements for additional thymus microenvironments during iNKT cell development are unknown. In this study, we reveal a role for medullary thymic epithelial cells (mTECs) during iNKT cell development in the mouse thymus. This requirement for mTECs correlates with their expression of genes required for IL-15 *trans*-presentation, and we show that soluble IL-15/IL-15Rα complexes restore iNKT cell development in the absence of mTECs. Furthermore, mTEC development is abnormal in iNKT cell–deficient mice, and early stages in iNKT cell development trigger receptor activator for NF-κB ligand–mediated mTEC development. Collectively, our findings demonstrate that intrathymic iNKT cell development requires stepwise interactions with both the cortex and the medulla, emphasizing the importance of thymus compartmentalization in the generation of both diverse and invariant αβT cells. Moreover, the identification of a novel requirement for iNKT cells in thymus medulla development further highlights the role of both innate and adaptive immune cells in thymus medulla formation.

## Introduction

In the thymus, immature CD4^+^CD8^+^ thymocytes expressing randomly generated αβTCRs undergo selection events to ensure self-MHC–restricted T cells are produced that are purged of autoreactivity (1). Thymus organization into cortical and medullary microenvironments underpins the multistage nature of αβT cell selection (2). In the cortex, positive selection via interactions with cortical thymic epithelial cells (cTECs) (3) triggers a differentiation program involving expression of CCR7, enabling newly selected cells to enter the medulla (4), where they interact with dendritic cells (DCs) and medullary TECs (mTECs), including the Aire^+^ subset (5, 6). In the medulla, negative selection eliminates autoreactive thymocytes via apoptosis and involves interactions with both DCs and mTECs (7). The importance of clonal deletion was recently emphasized in studies showing that escape of thymocytes from apoptosis resulted in T cell–mediated autoimmunity (8). Importantly, through mTEC expression of TNFR superfamily (TNFRSF) members such as receptor activator for NF-κB (RANK), CD40, and LTβR, thymus medulla formation depends on interactions with medullary-resident innate and adaptive immune cells (9), findings that highlight the importance of lymphostromal interactions in thymus medulla formation.

In addition to conventional αβ T cells, the thymus supports the development of invariant αβ T cell subsets, including CD1d-restricted invariant NKT (iNKT) cells (10). Classical iNKT cells, identified using CD1d tetramers loaded with α-galactosylceramide or its analog (PBS57) (11), predominantly express an invariant Vα14-Jα18 TCR and are implicated in controlling both innate and adaptive immune responses (12, 13). In contrast to conventional T cells, iNKT cells depend on recognition of CD1d/glycolipid complexes expressed by cortex-resident CD4^+^CD8^+^ thymocytes (14–17), a process that triggers a multistep maturation program including loss of CD24 and acquisition of CD44 and NK1.1 (10). Within the intrathymic CD1d/α-galactosylceramide tetramer^+^ iNKT cell pool, NK1.1^+^ cells include long-term thymic residents, the function of which is unclear (18). Interestingly, CXCR3 has been linked to thymic retention of these cells, with mTECs acting as a source of chemokine ligands (19), suggesting links between the medulla and intrathymic iNKT cells. Recent studies have also identified a retinoic acid–related orphan receptor (ROR)γt^+^IL-17^+^ iNKT cell subset that develops intrathymically via CD1d-mediated interactions (20). However, despite an understanding of intrathymic iNKT cell heterogeneity, as well as the processes controlling initiation of iNKT cell development in the cortex, the potential requirement for support from additional thymic microenvironments during iNKT cell development, as well as the possible impact they have on thymic microenvironments themselves, is unclear.

In this study, we show that mTECs play an important role during later stages of iNKT cell development in the thymus. Thymus transplantation experiments show that this requirement for mTECs does not correlate with their expression of the B7 family members CD80, CD86, and ICOSL, known regulators of iNKT cell development. Instead, we show that provision of soluble IL-15/IL-15Rα complexes at least partially restores iNKT cell development in the absence of mTECs, which maps to their expression of genes required for IL-15 *trans*-presentation. Finally, we reveal impaired development of the mTEC compartment in iNKT cell–deficient *CD1d^−/−^* mice and show that iNKT cells can stimulate RANK-dependent thymic medulla formation via Aire^+^ mTEC development. Collectively, we demonstrate a dual requirement for both cortical and medullary compartments during iNKT cell development in the thymus, and we reveal a novel role for iNKT cells in RANK-mediated thymus medulla formation.

## Materials and Methods

### Mice

*Relb^−/−^* (21), *Cd1d^−/−^* (22), *B7h^−/−^* (23), *B7-1/B7-2^−/−^* (24), and wild-type (WT) C57BL/6 mice were bred and maintained at the University of Birmingham. Embryonic mice were generated by timed pregnancies, and vaginal plug detection was designated day 0. Experiments were performed in accordance with University of Birmingham and U.K. Home Office regulations.

### Cell preparations

Thymocytes were recovered by mechanical disruption of isolated tissues, with analysis of thymic stromal cells performed following enzymatic disaggregation and depletion of CD45^+^ hemopoetic cells using microbeads (Miltenyi Biotec) as described (25).

### Abs and flow cytometry

Flow cytometry was performed using a BD LSRFortessa analyzer (BD Biosciences), whereas cell sorting was performed using an XDP cell sorter (Beckman Coulter), as described (26). The purity of isolated populations was typically >98%. The following were used (eBioscience unless otherwise stated): anti-CD4 allophycocyanin (FM4-5), anti-CD8 FITC (53-6.7), anti-TCRβ (H57-597), anti-NK1.1 PE/PE Cy7 (PK136), anti-CD24 PerCP Cy5.5 (M1/69), anti-CD44 A700/PE Cy7 (IM7), anti-RORγt PE (AFKJS-9), anti-CD16/32 (93), anti-CD45 allophycocyanin Cy7/allophycocyanin eFluor 780 (30-F11), anti-EpCAM1 allophycocyanin (G8.8), anti-Ly51 PE (6C3), anti–I-A^b^ biotin (Becton Dickinson, AF6-120.1), anti-Aire 488 (5H12), anti-ICOSL biotin (HK5.3), and anti-CD80 Brilliant Violet 421 (16-10A1; BioLegend). Biotinylated Abs were detected with streptavidin PE Cy7. Brilliant Violet 421/allophycocyanin–conjugated CD1d tetramers loaded with PBS57 were from the National Institutes of Health Tetramer Facility. For RORγt staining, cells were permeabilized with the Foxp3 staining kit (eBioscience). Aire staining (27) and staining using anti–RANK ligand (RANKL; IK22.5) and anti-CD40L (MR1; BD Biosciences) was performed as described (25).

### Quantitative PCR

Quantitative PCR analysis was performed as described (27). Primer sequences were: *Actb* (β-actin), QuantiTect Mm_*Actb*_1_SG primer assay (Qiagen; QT00095242); *Il15*, forward, 5′-TCTCCCTAAAACAGAGGCCAA-3′, reverse, 5′-TGCAACTGGGATGAAAGTCAC-3′; *Il15ra*, forward, 5′-CCCACAGTTCCAAAATGACGA-3′, reverse, 5′-GCTGCCTTGATTTGATGTACCAG-3′.

### Thymus transplantation and IL-15/IL-15Rα complex administration

2-Deoxyguanosine (dGuo)–treated fetal thymus organ culture (FTOCs) of the indicated strains were transplanted under the kidney capsule of 8- to 12-wk-old C57BL/6 mice (26). Soluble IL-15/IL-15Rα complexes were prepared as described (28). In brief, 2.5 μg rIL-15 (PeproTech) was mixed with 15 μg soluble IL-15Rα (R&D Systems) in 50 μl PBS and incubated at 30°C for 30 min. The volume was made up to 300 μl with PBS and injected i.p. into WT mice previously grafted with either WT or *Relb^−/−^* TECs. Grafts were recovered 4 d later and iNKT cell populations were analyzed by flow cytometry.

### Reaggregate thymus organ cultures

dGuo-treated FTOCs were used as a source of thymic stromal cells and mixed with purified thymic PBS57^+^ iNKT cells from C57BL/6 mice at a ratio of 1:1 as described (26). After 5 d, cultures were disaggregated with 0.25% trypsin and stromal elements and analyzed by flow cytometry.

## Results

### RelB-dependent mTECs are required for both RORγt^+^ and RORγt^−^ iNKT cell development

**FIGURE 1.**
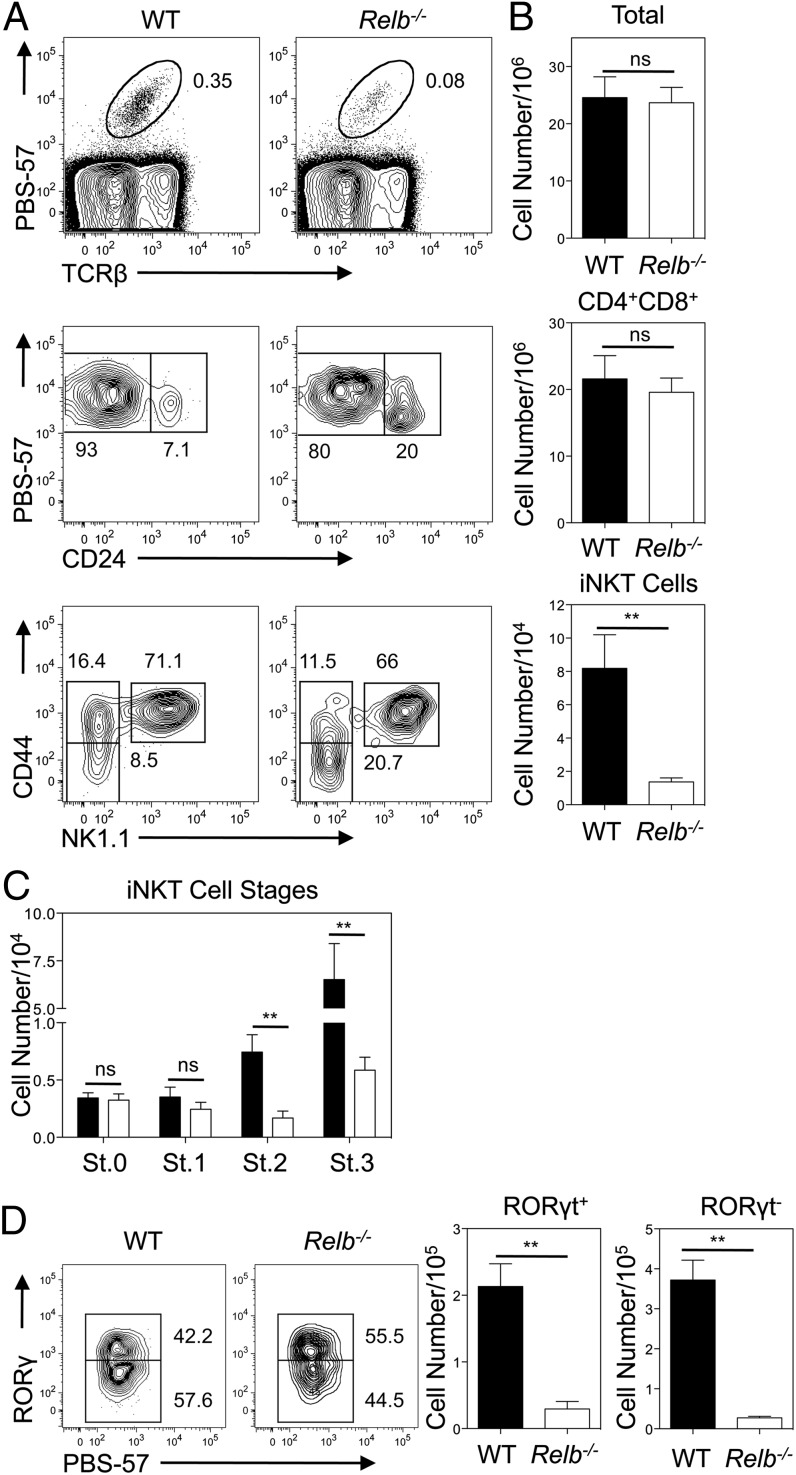
mTECs are required for intrathymic iNKT cell development. Thymocytes from WT and *Relb^−/−^* (**A**) were analyzed for iNKT cell development. (**B**) Absolute cell numbers of total thymocytes, CD4^+^CD8^+^ thymocytes, and CD1d/PBS57^+^ iNKT cells recovered from WT (filled bars) and *Relb*^−/−^ (open bars) thymus grafts, and numbers of cells at stages 0–3 of iNKT cell development are shown in (**C**). (**D**) Intracellular RORγt in CD1d/PBS57^+^ iNKT cells from WT and *Relb^−/−^* thymus grafts, as well as cell numbers of RORγt^+^ and RORγt^−^ iNKT cells in WT (filled bars) and *Relb^−/−^* (open bars) grafts. Error bars represent SEM. Data are from at least three separate experiments, in which groups of three mice were typically grafted with two to three thymus lobes. **p < 0.01 using a Mann–Whitney *U* test.

iNKT cell development initially involves interactions in the thymic cortex between CD4^+^CD8^+^ thymocytes. To investigate the potential involvement of additional thymic microenvironments during iNKT cell development, we made use of an experimental system in which *Relb^−/−^* TECs were transplanted under the kidney capsule of adult WT mice (26), whereby absence of RelB leads to a specific mTEC defect without the compounding immune defects of *Relb^−/−^* mice (21). Analysis of WT and *Relb^−/−^* TEC grafts recovered after 8 wk showed that despite comparable numbers of total and CD4^+^CD8^+^ thymocytes, *Relb^−/−^* TEC grafts contained a significant reduction in both the proportion and absolute cell number of PBS57/CD1d tetramer^+^ iNKT cells (Fig. 1A, 1B). Further analysis revealed a selective and significant reduction in absolute numbers of stage 2 (CD44^+^NK1.1^−^) and stage 3 (CD44^+^NK1.1^+^) subsets in *Relb^−/−^* TEC grafts (Fig. 1A, 1C). Thus, whereas induction of iNKT cell development occurs independently of mTECs, a finding in line with their initial selection by cortical thymocytes present in normal frequencies in *Relb^−/−^* TEC grafts (Fig. 1B), later stages of iNKT cell development require RelB-dependent mTECs. We next combined PBS57/CD1d tetramer^+^ staining with nuclear staining of RORγt to analyze the requirement for mTECs in the development of both RORγt^+^ iNKT17 and RORγt^−^ iNKT cells. Interestingly, whereas proportions of both RORγt^+^ and RORγt^−^ subsets within the total iNKT cell population were comparable in WT and mTEC-deficient *Relb^−/−^* grafts, absolute numbers of both showed a similar and significant reduction within *Relb^−/−^* grafts (Fig. 1D). Thus, RelB-dependent mTECs are required during the intrathymic development of both RORγt^+^ and RORγt^−^ iNKT cells.

### Soluble IL-15/IL-15Rα complexes partially restore iNKT cell development in the absence of the thymus medulla

**FIGURE 2.**
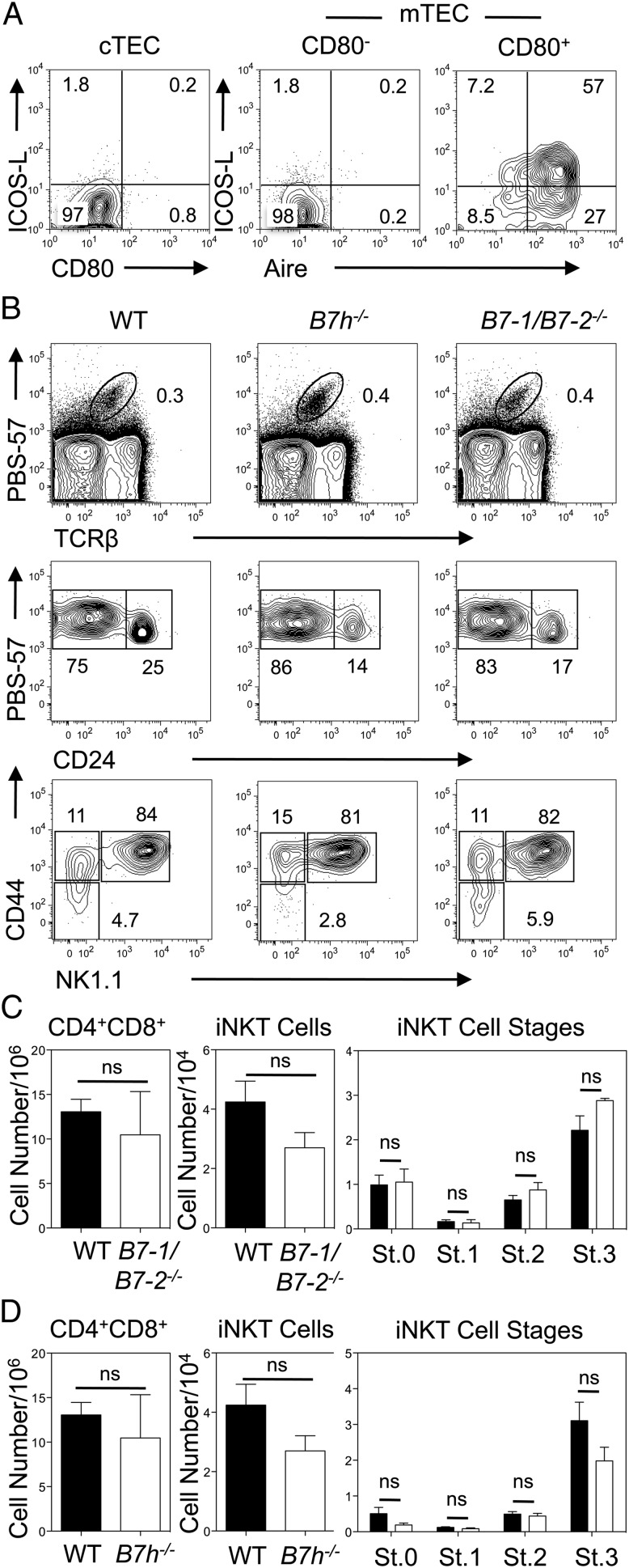
B7 family costimulation from mTECs is dispensable for iNKT cell development. (**A**) Analysis of ICOSL/CD80 expression in EpCAM1^+^CD45^−^Ly51^+^ cTECs, as well as ICOSL/Aire expression in CD80^−^ and CD80^+^ EpCAM1^+^CD45^−^Ly51^−^ mTECs. Thymocytes from WT, *B7h^−/−^*, and *B7.1/B7.2^−/−^* TEC grafts (**B**) were analyzed for iNKT cell development. (**C** and **D**) Absolute cell numbers of CD4^+^CD8^+^ thymocytes and total iNKT cells, together with stages 0–3 of iNKT cell development. Filled bars represent WT TEC grafts; open bars represent either *B7h^−/−^* or *B7.1/B7.2^−/−^* TEC grafts, as indicated. Error bars represent SEM. Data were obtained from two separate experiments, which included a minimum of two to three thymus grafts from groups of three mice.

The CD80/CD86/ICOSL members of the B7 family, either individually or in combination, represent important regulators of intrathymic iNKT cell development (29, 30). Interestingly, although several studies have reported CD80/CD86 expression by mTECs (31, 32), the expression of ICOSL within medullary compartments is less clear. Analysis of ICOSL in medullary thymic epithelium demonstrated that ICOSL is also expressed within the CD80^+^ mTEC compartment, including a proportion of Aire^+^ cells (Fig. 2A). However, given that CD80/CD86 and ICOSL are also expressed by intrathymic APCs of hemopoietic origin, it is unclear whether their expression by mTECs explains the requirement for these cells in iNKT cell development shown in the present study. To investigate this, we transplanted either CD80/CD86 double-knockout (*B7-1/B7-2^−/−^*) or ICOSL knockout (*B7h^−/−^)* TECs under the kidney capsule of WT mice, generating thymic microenvironments in which either CD80/86 or ICOSL expression was selectively absent from mTECs. Analysis of development in WT, *B7h^−/−^*, and *B7-1/B7-2^−/−^* TEC grafts showed no differences in the proportions (Fig. 2B) and absolute numbers of iNKT cell subsets (Fig. 2C), indicating that the requirement for mTECs does not map to expression of B7 molecules.

**FIGURE 3.**
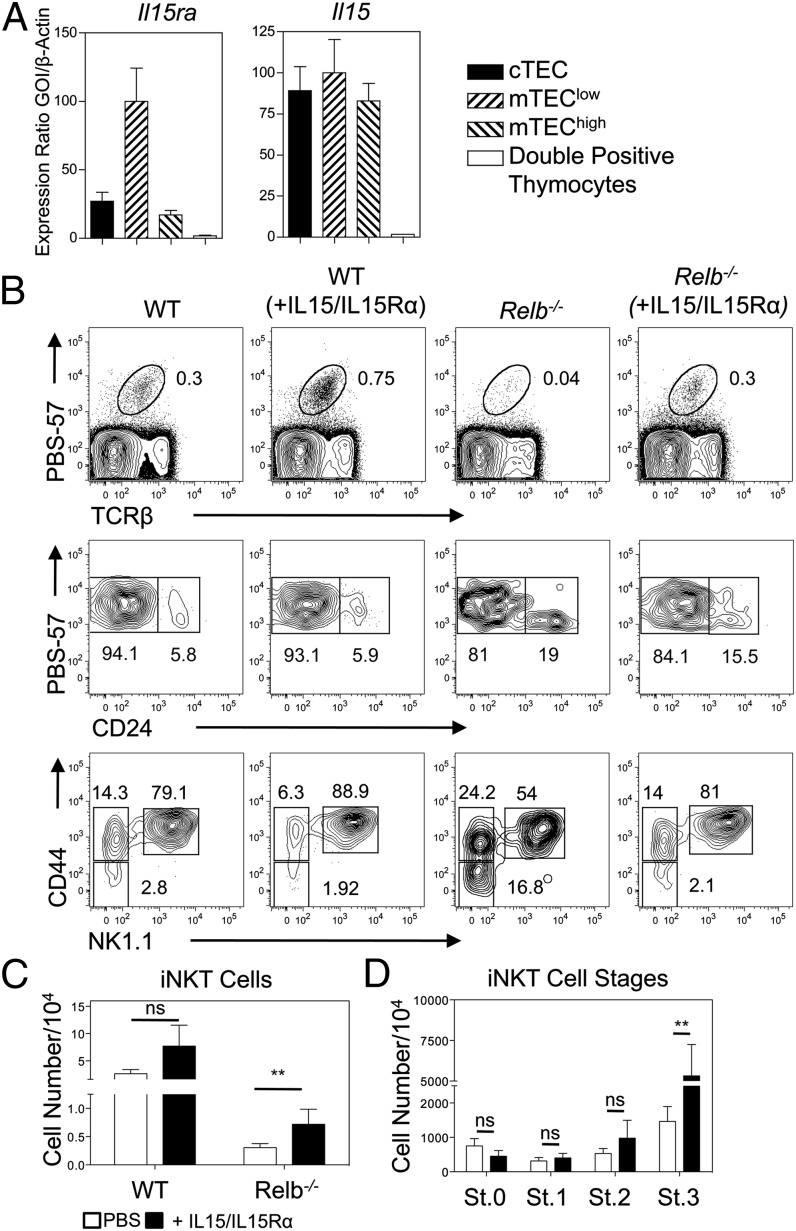
Soluble IL-15/IL-15Rα complexes enhance iNKT cell development in the absence of an intact medulla. (**A**) Quantitative PCR of the indicated WT adult TEC populations for *Il15* and *Il15rα* mRNA. Levels of mRNA were normalized to β-actin. Error bars represent SEM, and data are representative of at least two independently sorted biological samples, with each gene analyzed in triplicate. (**B**) iNKT cell development in mice receiving either WT or *Relb*^−/−^ TEC grafts, injected with soluble IL-15/IL-15Rα complexes, as indicated. (**C**) Absolute cell numbers of total iNKT cells in mice receiving WT or *Relb*^−/−^ TEC grafts without (open bars) or with (filled bars) IL-15/IL-15Rα treatment. (**D**) Absolute numbers of iNKT cells in mice receiving *Relb*^−/−^ TEC grafts without (open bars) and with (filled bars) IL-15/IL-15Rα treatment. Data in (B)–(D) are from three separate experiments, with a minimum of three grafts from thre mice per group. Error bars represent SEM. **p < 0.01 using a Mann–Whitney *U* test.

Intrathymic iNKT cell development is also known to depend on availability of IL-15, a cytokine which is *trans*-presented via the high-affinity IL-15Rα to responding IL-15Rβ/γc–expressing cells (28, 33). Interestingly, consistent with our observations in mTEC-deficient thymus grafts (Fig. 1), numbers of iNKT cells at later stages of development are notably reduced in both *Il15^−/−^* and *Il15ra^−/−^* mice (33–35). To investigate whether provision of IL-15/IL-15Rα is linked to the requirement for mTECs in iNKT cell development, we performed analysis of *Il15* and *Il15ra* gene expression in purified TEC subsets. Whereas *Il15* mRNA is readily detectable in multiple TEC populations, including cTECs, MHC class II (MHC II)^low^ and MHC II^high^ mTEC subsets, *Il15ra* mRNA was most abundant in the MHC II^low^ mTEC subset (Fig. 3A). Thus, the MHC II^low^ mTEC compartment selectively expresses both genes necessary for the production and *trans*-presentation of IL-15. To determine whether provision of soluble IL-15/IL-15Rα complexes enhanced iNKT cell development in mTEC-deficient TEC grafts, we grafted WT mice with either WT or *Relb^−/−^* TEC grafts, and after 8 wk injected them with soluble IL-15/IL-15Rα complexes. Four days after injection, treatment with IL-15/IL-15Rα complexes was found to have caused an increase in the proportion of PBS57^+^ iNKT cells and a nonsignificant increase in the iNKT cell numbers in WT grafts (Fig. 3B, 3C). Strikingly, IL-15/IL-15Rα treatment of mice carrying *Relb*^−/−^ TEC grafts caused a significant increase in iNKT cells within the grafted thymus. Thus, although iNKT cell numbers in *Relb*^−/−^ thymuses increased ∼2-fold following IL-15/IL-15Rα treatment (Fig. 3C), iNKT cell proportions (Fig. 3B) significantly increased 10-fold (noninjected *Relb*^−/−^ grafted mice 0.03% iNKT cells, SEM ± 0.03%; IL-15/IL-15Rα–injected *Relb*^−/−^ grafted mice 0.3% iNKT cells, SEM ± 0.05%, *p* < 0.01). Moreover, analysis of iNKT cell subsets in *Relb*^−/−^ thymus-transplanted mice showed that IL-15/IL-15Rα stimulation selectively increased the numbers of later stages of iNKT cell development, whereas early developmental stages 0 and 1 were unaltered (Fig. 3D). Interestingly, in vivo IL-15/IL-15Rα stimulation of *Relb*^−/−^ thymuses did not fully restore iNKT cells to numbers observed within untreated WT grafts (Fig. 3C). The reason for this is not clear, although it could reflect either the short (4 d) time span of IL-15/IL-15Rα stimulation, or indicate that mTECs play additional roles in iNKT cell development and/or expansion that are distinct from their provision of IL-15/IL-15Rα. Whatever the case, these data suggest that production of IL-15/IL-15Rα by mTECs at least partly explains their involvement during later stages of iNKT cell development.

### Developing iNKT cells express RANKL and induce the development of Aire^+^ mTECs

**FIGURE 4.**
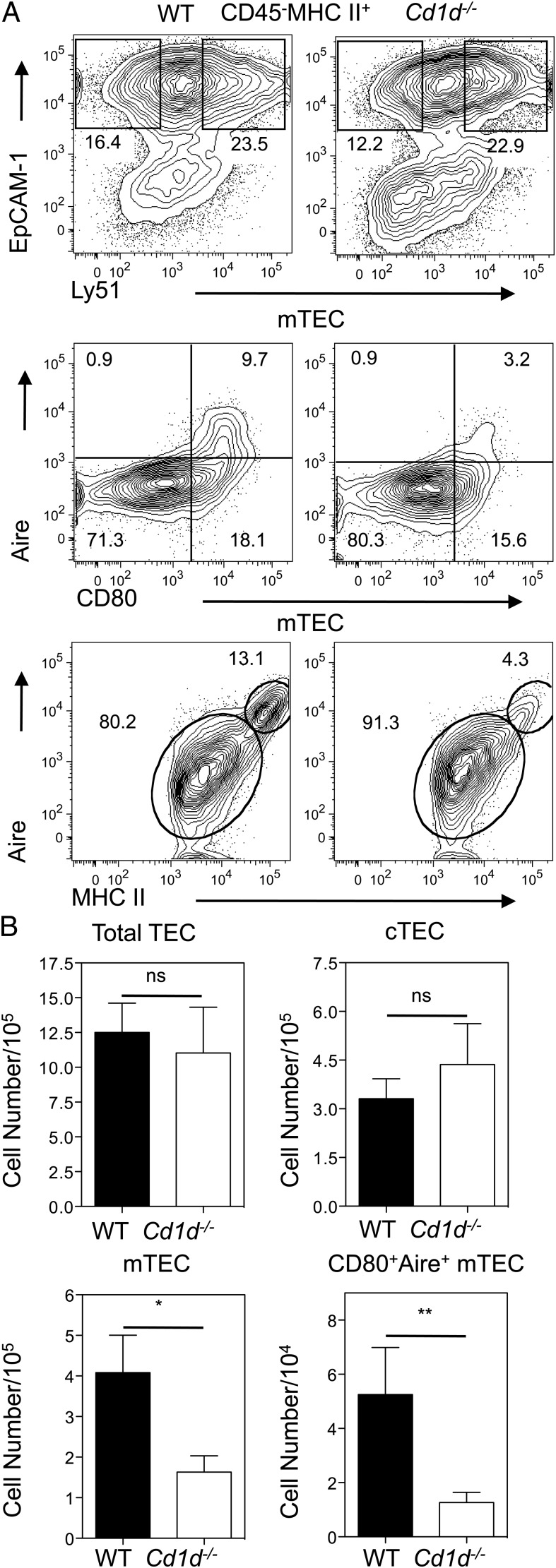
Aire^+^ mTECs are selectively reduced in *Cd1d^−/−^* mice. Thymic stromal preparations from adult WT and *Cd1d*^−/−^ mice were digested and the CD45^−^ MHC II^+^ stromal compartment was analyzed for the frequencies of Ly51^+^EpCAM1^+^ cTECs and Ly51^−^EpCAM1^+^ mTECs. Subsets of mTECs were defined by CD80, MHC II^low^, MHC II^high^, and Aire (**A**). (**B**) Absolute cell numbers of the indicated populations in WT (filled bars) and *CD1d^−/−^* (open bars) mice. Error bars represent SEM. Data are from three separate experiments, with a minimum of three mice per group. *p < 0.05, ** *p* < 0.01 using a Mann–Whitney *U* test.

**FIGURE 5.**
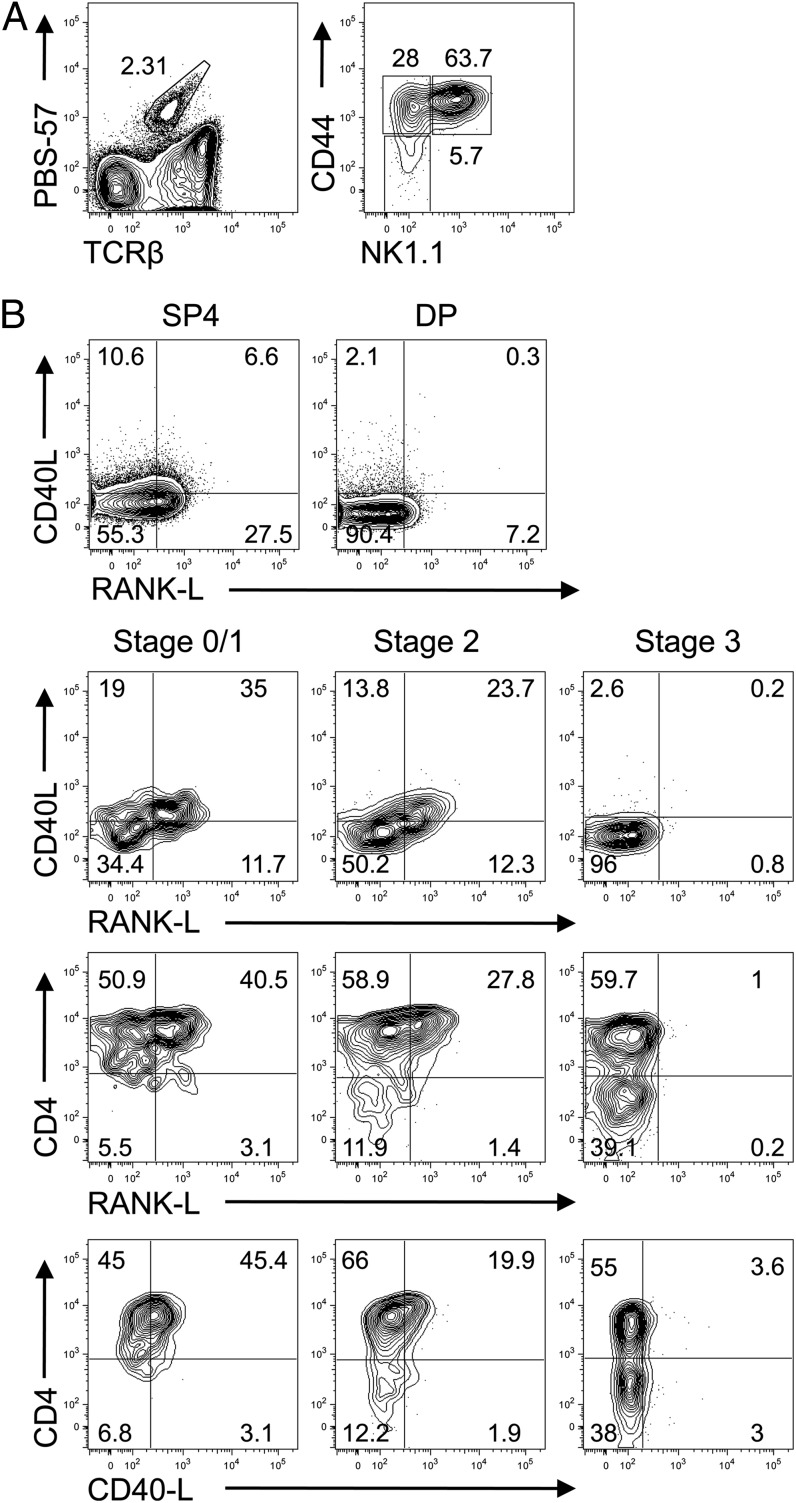
RANKL is expressed during early stages of intrathymic iNKT cell development. (**A**) Freshly isolated adult thymocytes stained with CD1d/PBS57 tetramers together with Abs to TCRβ, CD44, NK1.1, after gating on CD8^−^ cells. (**B**) *Upper panels*, RANKL/CD40L expression on CD4^+^CD8^+^ and CD4^+^CD8^−^ CD1d/PBS57 tetramer^−^ thymocytes; *lower panels*, RANKL/CD40L on the indicated subsets of thymic iNKT cells. Note the expression of RANKL and CD40L during stages 0–2, but not stage 3, of iNKT cell development. Data shown are representative of at least three independent experiments.

**FIGURE 6.**
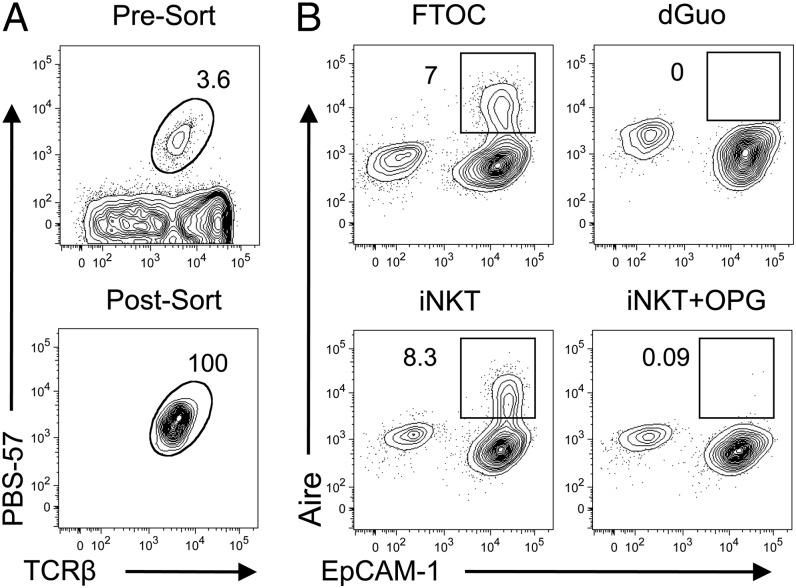
iNKT cells induce RANK-mediated Aire^+^ mTEC development. FACS-purified PBS57/CD1d^+^ iNKT cells from adult thymus (**A**) were incorporated into RTOCs at a 1:1 ratio with dGuo thymic stroma in the presence or absence of recombinant osteoprotegerin. Presort sample in (A) is after gating on CD8^−^ cells. (**B**) Disaggregated cell suspensions obtained 5 d after culture and analyzed for expression of EpCAM1 and intracellular Aire. Data shown are gated on CD45^−^Ly51^−^ stromal cells. Unmanipulated FTOCs and dGuo stroma alone are shown for comparison. Data are typical of at least three independent experiments.

Whereas the adult thymus supports the development of invariant αβTCR^+^ iNKT cells, the fetal thymus supports the development of multiple waves of invariant γδT cells. Given our recent findings highlighting the impact of fetal invariant γδT cells on thymus medulla formation (27), we investigated the potential involvement of iNKT cells in this process. To investigate whether iNKT cells influence mTEC development in vivo, we analyzed the thymic stromal compartment of adult WT and *Cd1d^−/−^* mice. Interestingly, we observed a selective and significant reduction in the proportion and absolute numbers of mTECs in *Cd1d^−/−^* mice, including the more mature CD80^+^Aire^+^ subset (Fig. 4). Thus, Cd1d-dependent iNKT cells are required for normal thymus medulla development in vivo. Moreover, when we analyzed stages in iNKT cell development for expression of CD40L and RANKL, representing two TNFRSF ligands linked to mTEC development (31, 36, 37), both were expressed at stages 0–2 of development but were absent from stage 3 CD44^+^NK1.1^+^ cells (Fig. 5). Thus, intrathymic development of iNKT cells involves temporal upregulation of TNFSF molecules that drive mTEC development. To directly test the potential of thymic iNKT cells to play a role in this process, we incorporated freshly purified PBS57 tetramer^+^ thymic iNKT cells (Fig. 6A) into reaggregate thymus organ cultures (RTOCs) prepared from alymphoid dGuo-treated fetal thymus lobes devoid of mature CD80^+^MHC II^high^Aire^+^ mTECs (31). In contrast to RTOCs containing dGuo-treated stromal cells alone (Fig. 6B), RTOCs receiving purified thymic PBS57/CD1d tetramer^+^ iNKT cells contained a readily detectable population of mature mTECs expressing Aire (Fig. 6B), similar to that observed in unmanipulated lymphoid FTOCs. Furthermore, despite their expression of CD40L (Fig. 5B), addition of recombinant osteoprotegerin, a soluble decoy receptor for RANKL, completely abrogated the ability of iNKT cells to stimulate Aire^+^ mTEC development (Fig. 6B). Collectively, these findings reveal a novel role for iNKT cells in medulla formation, as demonstrated by mTEC abnormalities in iNKT cell deficient *CD1d^−/−^* mice, as well as the ability of RANKL^+^ iNKT cells to stimulate mTEC development in vitro.

## Discussion

In addition to its role in fostering the generation of “mainstream” CD4^+^ and CD8^+^ αβT cells, the thymus is also a site for production of nonconventional T cell populations, including multiple waves of invariant γδT cells during fetal life and invariant CD1d-restricted NKT cells in the postnatal thymus. In this study, we have investigated the thymic microenvironmental requirements of CD1d-resticted iNKT cells and examined the impact of these cells on thymus medulla development. We show that normal development of both RORγt^−^ and RORγt^+^ “iNKT17” (20) cells depends on the presence of mTECs, indicating common microenvironmental requirements for the development of discrete iNKT cell populations. Interestingly, earlier experiments showed that RelB^+^ radioresistant cells are also involved in iNKT cell development. However, the precise identity of the RelB^+^ cell, as well as the specific requirement for an intact mTEC compartment during iNKT cell development, has not been fully addressed (38, 39). Collectively, we suggest that stepwise developmental events first in the cortex and then the medulla regulate iNKT cell development. Further thymus transplantation experiments were also performed to study the molecular basis of mTEC involvement in iNKT cell development. Whereas mTEC expression of CD80/86 or ICOSL was found to be dispensable for iNKT cell development, administration of soluble IL-15/IL-15Rα complexes at least partially substituted for the absence of mTECs in *Relb^−/−^* thymus grafts. Specifically, a significant increase in the numbers of stage 3 iNKT cells was observed, whereas stage 2 iNKT cells showed a similar trend, despite a lack of significance. That combined expression of *Il15* and *Il15ra* mRNA is limited to mTEC^low^ suggests a role for this mTEC subset in the IL-15–mediated expansion of iNKT cells following their generation in the cortex. Finally, that the mTEC^low^ compartment is functionally involved during iNKT cell development may be of particular significance, given that this subset has recently been shown to express molecules of functional importance for conventional αβT cells, including CCL21, to attract positively selected CCR7^+^ thymocytes to medullary regions (40). Thus, the mTEC^low^ compartment, as well as containing progenitors of mTEC^high^ (31, 32) and stages of mTEC development after Aire expression (41), is functionally linked to the maturation of various intrathymic αβT cell subsets.

**FIGURE 7.**
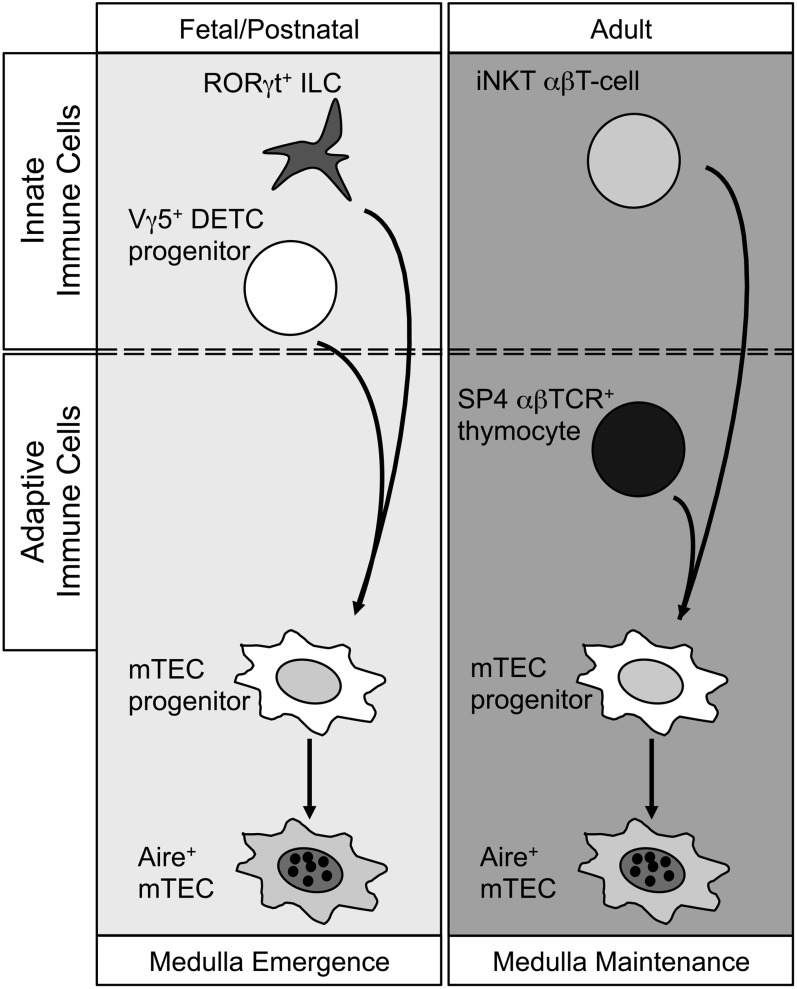
A model for the establishment and maintenance of Aire^+^ medullary thymic microenvironments. In both fetal and adult stages, signals through the TNFRSF member RANK play a key role in inducing the development of mTEC progenitors to the Aire^+^ stage. mTECs expressing Aire emerge around embryonic day 16 of gestation, and both RORγt^+^ innate lymphoid cells and Vγ5^+^ dendritic epidermal T cell progenitors influence the generation of the first cohorts of Aire^+^ mTECs, at stages prior to αβT cell positive selection. In adult stages, RANKL expression from positively selected CD4^+^CD8^−^ thymocytes, as well as intrathymically generated iNKT cells, ensures the continued production of Aire^+^ mTECs.

Additionally, we show that thymic iNKT cells play a role in the development of Aire^+^ mTECs via a mechanism involving RANKL-mediated triggering of mTEC progenitors. Such a finding is also supported by reduced mTEC development in the adult thymus of *Cd1d^−/−^* mice. Interestingly, whereas γδT cells are dispensable for adult thymus medulla formation (37), invariant Vγ5^+^ dendritic epidermal T cell progenitors stimulate RANK-dependent mTEC development in the fetal thymus (27). Collectively, these findings support a model in which the impact of invariant T cells on thymic medulla formation involves a temporal sequence of invariant γδ T cells and then invariant αβ T cells during fetal and adult stages, respectively (Fig. 7). In this model, the establishment of the Aire^+^ mTEC compartment in fetal life, which is initiated around embryonic day 16 of gestation (42), involves interactions with both RORγt^+^-expressing innate lymphoid cells and invariant Vγ5^+^ dendritic epidermal T cell progenitors. That the presence of Aire^+^ mTECs at the neonatal stage is essential in purging the nascent αβ T cell reprtoire of autoreactive specificities (5) perhaps highlights the importance of this initial control of thymus medulla development for tolerance induction. Additionally, the continued production of Aire^+^ mTECs as a consequence of mTEC turnover in adult life also involves input from invariant (iNKT) and diverse (CD4 αβ T cells) cell types, again highlighting interplay between the innate and adaptive immune systems.

Analysis of RANKL expression in developmentally distinct stages of iNKT cells suggests that their ability to induce Aire^+^ mTECs occurs prior to the acquisition of NK1.1, representing early developmental stages arising shortly after their positive selection on CD1d molecules in the cortex. Interestingly, we recently described a similar pattern of RANKL expression during the early (CD69^+^) stages in conventional CD4^+^ thymocyte development (25, 37), suggesting that the ability of both MHC II–restricted and CD1d-restricted thymocytes to induce Aire^+^ mTEC development occurs shortly after the entry of these cells into the thymic medulla. In addition to newly generated iNKT cells, the thymus also contains NK1.1^+^ iNKT cells that persist intrathymically for as long as 1 y (18), a process linked to expression of CXCR3 and mTEC production of CXCL10 (19). However, absence of RANKL from NK1.1^+^ iNKT cells argues against the idea that thymic retention of iNKT cells contributes to intrathymic availability of RANKL for continued Aire^+^ mTEC development. Thus, the possible importance of thymic-resident iNKT cells remains unclear.

In conclusion, we reveal a role for mTECs during later stages of iNKT cell development and provide evidence for a stepwise intrathymic iNKT cell development program mediated by first cortical and then medullary thymus regions. That such microenvironmental requirements are shared by additional thymus-dependent T cell lineages further emphasizes the functional importance of thymus compartmentalization for T cell development, whereas a reciprocal requirement for iNKT cells during RANK-mediated thymic medulla development extends our understanding of the crosstalk mechanisms controlling mTEC development.
